# Mole on Face

**Published:** 1891-06

**Authors:** 


### MOLE ON FACE.

A hairy mole which is still growing should be removed at once, even at the risk of some injury to the skin. It is now probably no more than a plexus of capillary vessels, with only a small supply of connective tissue. There is also a likelihood that it has not yet involved the skin. If this be its condition, the skin over the tumor may be reflected in flaps, and the tumor itself strangulated with ligature in one of the usual ways; the flaps should then be replaced, and the result will be a minimum of cicatrix and deformity. But if the nævus be allowed to grow, it will become a large, highly vascular, erectile tumor, probably invading and involving the skin, liable to profuse hemorrhage if injured; yet still quite amenable to treatment, though of a less simple kind. The modes of treating nævi are numerous, and are continually increasing.

